# *In silico* design and *in vitro* validation of a multi-epitope peptide vaccine targeting triple-negative breast cancer

**DOI:** 10.3389/fonc.2025.1611991

**Published:** 2025-06-23

**Authors:** Pengjun Zhou, Xing Shi, Jinquan Xia, Hong Hu

**Affiliations:** ^1^ Department of Pharmacology, Guangdong Pharmaceutical University, Guangzhou, China; ^2^ Beijing Institute of Genomics, Chinese Academy of Sciences, Beijing, China; ^3^ Department of Clinical and Research Center, Shenzhen People’s Hospital (The First Affiliated Hospital, Southern University of Science and Technology; The Second Clinical Medical College, Jinan University), Shenzhen, Guangdong, China; ^4^ Division of Breast Surgery, Department of General Surgery, Shenzhen People’s Hospital (The First Affiliated Hospital, Southern University of Science and Technology; The Second Clinical Medical College, Jinan University), Shenzhen, Guangdong, China

**Keywords:** adjuvant, epitope, T-lymphocytes, toll-like receptor, triple-negative breast cancer (TNBC), *in vitro* analysis

## Abstract

**Aims and objectives:**

This study aimed to identify immunodominant epitopes from a panel of triple-negative breast cancer (TNBC)-associated proteins—MZF-1, Mucin-1, SOX-9, Keratin 5, Keratin 14, Twist1, and Progranulin (GP88)—to design multi-epitope peptide vaccines capable of eliciting robust anti-tumour immune responses.

**Methods:**

A comprehensive immunoinformatics pipeline was employed. Amino acid sequences were retrieved from UniProt and analysed to predict CTL, HTL, B-cell, and IFN-γ-inducing epitopes. Top candidates were filtered based on antigenicity, allergenicity, glycosylation, and HLA coverage. Molecular docking was conducted with HLA alleles to assess binding affinity. Five multi-epitope vaccine constructs were designed using different adjuvants (GM-CSF, β-defensin, IL-2, cholera enterotoxin, and 50S ribosomal protein L7/L12), and enhanced with PADRE and HEYGAEALERA sequences. Structural modelling, refinement, disulfide engineering, and validation (via Robetta, GalaxyRefine, ProSA, and Ramachandran plots) were performed, followed by docking with TLR2 and TLR4. Immune simulation assessed cytokine responses and memory generation. *In-vitro* validation using MDA-MB-231 cells tested immunostimulatory activity of top-ranked CTL peptides.

**Results:**

Thirteen CD8+ CTL, thirteen CD4+ HTL, and seven B-cell epitopes were selected based on favourable immunogenic properties and high HLA promiscuity. Constructs V1 (GM-CSF-linked) and V5 (β-defensin-linked) exhibited superior TLR2/4 docking affinity. Immune simulation showed V2 and V5 induced strong cytokine release and memory cell responses. *In vitro* assays demonstrated enhanced expression of MZF-1, SOX-9, and Twist1, confirming epitope-driven immune activation.

**Conclusion:**

This study successfully identified potent immunogenic epitopes from TNBC-associated proteins and constructed promising multi-epitope vaccines. Constructs V1 and V5 demonstrated superior immunogenicity and TLR binding, while V2 and V5 induced strong immune responses *in silico*. These findings provide a foundation for developing effective peptide vaccines against TNBC.

## 1 Introduction

Triple-negative breast Cancer (TNBC) stands out as a highly aggressive form of breast cancer distinguished by the lack of expression of certain receptors, namely human epidermal growth factor receptor 2 (HER2), estrogen, and progesterone (1). Due to the absence of these receptors, TNBC remains refractory to most of the available targeted therapies, rendering it a formidable challenge in oncology (2). Notably, this subtype of breast cancer displays an increased propensity for metastasis, frequently leading to worse patient outcomes when compared to other subtypes of breast cancer. The chemotherapy with drugs like anthracyclines and taxanes is considered standard treatment for TNBC. For patients with PD-L1-positive TNBC, the checkpoint inhibitors are added. However, it has been earlier been demonstrated that it has not worked well in some patients, showing challenges like immune escape, resistance and side effects. Other treatments like PARP inhibitors and antibody-drug conjugates have also shown limited usefulness due to toxicity and resistance problems (3). Hence, there is a pressing need to develop effective therapeutic strategies for TNBC. Immunotherapy holds promise when compared to chemotherapy and radiation therapy in cancer treatment (4). Recent advancements in the field of immuno-informatics have unveiled opportunities to identify potent immunotherapeutic targets against various cancers, including TNBC. Through its computational capabilities, immuno-informatics can aid in the identification and design of potential vaccine targets with high precision and efficacy, minimising the time-consuming and costly experimental setups (5). Since TNBC represents greater genetic instability, multiepitope-guided immune therapeutics hold promise (6). Previously, several research groups have proposed multiepitope-based vaccines against different cancers (7), viral (8), bacterial (9), fungal (10), and parasitic pathogens (11), and several other non-communicable diseases as well. Even most of the clinical trials of peptide-based therapeutics are on breast cancer, followed by other cancers (12). In view of the growing significance of peptide-based therapeutics, the identification and characterisation of the potential peptides or epitopes with therapeutic efficacy is of paramount importance. Recognising this importance, our study has strategically targeted several proteins like MZF-1, Mucin-1, SOX-9, Basal-like antigens (Keratin 5 and Keratin 14), Twist-related protein-1, and Progranulin (GP88), which are known to be associated with TNBC. Further, we aimed at identifying the immunogenic regions, i.e. epitopes in these proteins that may aid in developing an epitope cocktail-based vaccine. Myeloid Zinc Finger-1 is a transcription factor recognised for its role in governing diverse cellular processes such as proliferation, differentiation, and apoptosis. Some studies have indicated its overexpression in TNBC, suggesting its role in tumour progression and metastasis. Targeting MZF-1 could disrupt the transcriptional regulation of genes essential for TNBC growth and survival (13). Mucin-1 (MUC1) is a glycoprotein that is aberrantly glycosylated and overexpressed in many cancers, including TNBC. Its overexpression has been associated with increased invasiveness, metastasis, and resistance to chemotherapy in TNBC. Given its accessibility on the cell surface and its role in tumour progression, MUC1 also presents a potential therapeutic target (14). SOX-9, a transcription factor, has been shown to play a crucial role in managing the complex phenomenon of epithelial-to-mesenchymal transition (EMT), which constitutes a pivotal event in the metastatic progression of cancer. Its upregulation in TNBC signifies its involvement in the aggressive nature of this cancer subtype and underscores its potential as a therapeutic target (15). Another antigen, Basal-like breast cancers, which include a majority of TNBCs, often express specific markers such as Cytokeratin-5 and Cytokeratin-14. These antigens, being specific to a subset of TNBC, could serve as potential targets for immunotherapy, providing a more tailored approach to treatment (16). TWIST1 is a transcription factor involved in EMT. Its expression in TNBC has been correlated with increased metastasis, drug resistance, and poor prognosis. Targeting TWIST1 could potentially hinder TNBC’s metastatic capabilities and enhance the effectiveness of other therapies (17). Progranulin, or GP88, is known to be involved in cell proliferation, migration, and survival. Its overexpression in TNBC has been linked to enhanced tumour growth, metastasis, and resistance to apoptosis. Given its multifaceted role in tumour progression, GP88 also serves as an attractive candidate for therapeutic targeting in TNBC (18). As discussed, the proteins selected as potential targets for a vaccine against TNBC in the present study are due to their important roles in tumour growth, progression, and metastasis. Targeting these specific proteins could provide a multi-pronged approach in treating TNBC, potentially enhancing treatment outcomes and survival rates. An immuno-informatics approach was utilised to scrutinise these proteins for identifying the immunogenic regions that may be employed for vaccine development.

## 2 Materials and methods

All *in-silico* experiments, including epitope prediction, structural modelling, and immune simulations, were conducted using publicly available datasets and computational tools and thus did not involve the use of live animals or human participants, requiring no specific ethical approval. The *in-vitro* validation experiments were conducted in accordance with the institutional biosafety and ethical guidelines. These cells were obtained from a certified repository and cultured under sterile conditions in a biosafety level 2 (BSL-2) facility. No human or animal subjects were directly involved in this study, and thus no informed consent or animal ethics approval was required.

### 2.1 Protein targets

The amino acid sequences of the target proteins MZF-1, Mucin-1, SOX-9, Basal-like antigens (K5 and K14), Twist-related protein-1, and Progranulin (GP88) were retrieved from UNIPROT database with IDs P28698, Mucin-1, P48436, P13647 & P02533, Q15672, and P28799 respectively. The 3D models of TLR-2, TLR-4, HLA-A*02:01, DRB1 *01:01 and DRB1 *1501 were obtained from the Protein Data Bank (PDB) with the following identifiers: 6nig, 2z63, 1QEW, 2g9h and 1bx2, respectively.

### 2.2 Identification of epitopes (HTLs, CTLs, IFN‐γ and B cells)

The amino acid sequences of all target proteins underwent screening for HTL and CTL epitopes. Specifically, the sequences were examined for the presence of epitopes targeting 16 HTLs (with >95% of the worldwide population) and 12 CTLs (with >90% of the worldwide population), to cover the maximum worldwide population. The HTL epitopes were predicted by recently updated NetMHCIIpan 4.1 EL and NetMHCII pan 4.1 BA methods (19). In NetMHCII-pan versions 4.0 and beyond, distinct predictors have been introduced for binding (BA) and elution (EL). The binding predictions evaluate the peptide’s capability to bind to HLAs, while elution predictions go beyond by evaluating the probability of the peptides undergoing natural processing and presentation to HLA. This additional consideration enhances the likelihood of the peptide being recognised as a T-cell epitope. Based on the percentile ranks, the HTL epitopes were classified into three groups: strong, intermediate and non-binders. The peptides with percentile ranks ≤10 were considered as strong binders, between 11–50 as weak binders and >50 as non-binders. The CTL epitopes were predicted by combined methods: NetChop/NetCTL/NetCTLpan for effective cleavage (proteasomal processing) and T-cell epitope identification (20–22). A dual approach incorporating motif analysis and support vector machine (SVM) was utilised, employing a scanning module for the prediction of IFN‐γ epitopes through the IFN-epitope server. The predictive model was designed to distinguish between IFN‐γ versus Non‐IFN‐γ as discussed by Tau and Rothman (23). B cell epitope prediction was performed utilising three different tools, namely BepiPred-2.0, ABCPred and BcPred servers (24–26). Furthermore, structural and biochemical factors were also taken into consideration for evaluating the forecasted linear B-cell epitopes. The length of epitopes for CTL, HTL and B cell epitopes was set at 9mers, 15mers and 16mers, respectively.

### 2.3 Refining predicted epitopes through immune filters

Integrating widely adaptable epitopes plays a crucial role in designing vaccines, given their ability to bind with various HLA alleles. This versatility ensures comprehensive population coverage, making them a valuable component in vaccine development (27). In the present study, we have selected only those epitopes which were promiscuous in nature, having affinity towards multiple HLT and CTL alleles.

### 2.4 Evaluation of the epitope’s antigenicity and allergenicity

The antigenicity and allergenicity of the epitope were assessed through the utilisation of the AlgPred and VaxiJen v2.0 tools. The application of the SVM module based on amino acid composition was conducted to predict allergenicity, employing a threshold value of -0.4. This threshold is reported to have high sensitivity and specificity of 88.87% and 81.86% through fivefold cross‐validation. This specific approach in AlgPred showcased the highest sensitivity, leading to its selection for the analysis (28).

### 2.5 Analysis of post-translational modification sites within target proteins

The anticipated epitopes were examined to ascertain their occurrence within PTM sites using the NetOGlyc 4.0 server at http://www.cbs.dtu.dk/services/NetOGlyc/. Epitopes identified within these posttranslational modification sites were excluded from further examination.

### 2.6 Peptide and human leukocyte antigen interaction analysis

The investigation of spatial arrangements and binding configurations between epitopes (both HTL and CTL) and their corresponding HLA alleles was conducted using the ClusPro protein-protein docking server, developed by Schrödinger https://pymol.org/. The epitopes showing higher affinity to HLAs were selected.

### 2.7 Multiepitope chain construction, structure modelling, validation and physicochemical characteristics

The epitopes specific to cytotoxic T lymphocytes (CTLs), helper T lymphocytes (HTLs), and B cells were combined to formulate a multi-epitope vaccine. AAY linkers were used to connect CTL epitopes, GPGPG linkers for HTL epitopes, and KK linkers for B cell epitopes. Additionally, an adjuvant, PADRE sequence and HEYGAEALERA sequence were also linked to the multi-epitope construct. Five distinct vaccine constructs (V1-V5) were generated, each linked to a different adjuvant. The epitopes were randomly shuffled in five different ways to find the one with the best structural configuration. Robetta server (https://robetta.bakerlab.org/), an online homology modelling tool, was used for 3D modelling of the vaccine constructs, and was subsequently refined using the GalaxyRefine server (https://galaxy.seoklab.org/cgi-bin/submit.cgi?type=REFINE). The validation of the tertiary structure of the refined vaccine constructs was carried out using the ProSA webserver https://prosa.services.came.sbg.ac.at/prosa.php, ensuring that their overall quality fell within the range of z scores characteristic of native proteins (29). In order to enhance the evaluation of the quality of the models generated, a Ramachandran plot analysis was carried out using the MolProbity server https://phenix-online.org/documentation/reference/molprobity_tool.html. Further, disulfide engineering was carried out in order to improve the thermostability of the vaccine construct by Design 2 server (30). The confirmation of the physicochemical properties of the vaccine constructs was carried out through ProtParam https://web.expasy.org/protparam/, while the immunogenicity was assessed using the VaxiJen tool https://www.ddg-pharmfac.net/vaxijen/. Furthermore, two distinct servers, AlgPred and AllerTOP v2.0 tools, were employed to assess the allergenicity of the vaccine sequence. Lastly, the IEDB population coverage analysis was employed to evaluate the population size covered by the incorporated epitopes in the vaccine construct (31).

### 2.8 Analysis of interaction between vaccine models (V1 to V5) and immune receptors (TLRs)

The analysis of interaction patterns between the vaccine constructs and TLRs utilised ClusPro, a specialised tool for protein-protein docking (32). The models exhibiting the optimal docking configuration were finally selected.

### 2.9 Immune simulation of V2 and V5 vaccines

*In-silico* immune simulation was performed to assess the immune response triggered by the vaccine constructs utilising the C‐ImmSim server https://wwwold.iac.rm.cnr.it/~filippo/projects/c-immsim-online.html, which utilises a position‐specific scoring matrix. This server simulates the functions of critical immune components, including lymph nodes, thymus, and bone marrow, for the effective operation of the immune system (33). Simulation of different immune components belonging to innate, humoral and adaptive immune systems can be tested by the target sequence. Different parameters like the time set of injections, random seed and simulation steps were set.

### 2.10 *In vitro* validation

To validate the immunomodulatory potential of predicted epitopes, MDA-MB-231 cells (ATCC^®^ HTB-26™) were cultured in DMEM supplemented with 10% FBS. Cells were treated with synthetic peptides corresponding to the top-scoring CTL epitopes (10 µg/mL for 48 hours). Total RNA was extracted using TRIzol^®^, and cDNA was synthesised with the PrimeScript™ RT reagent kit (Takara Bio). qPCR was performed using SYBR Green Master Mix (Applied Biosystems) with primers specific for: MZF-1 (F: 5′-CAGGTGCTGAAGGACCTCTG-3′; R: 5′-TGGTGGTGTTGAGGTCATCA-3′); SOX-9 (F: 5′-AGGAAGCTCACGGAGCTCAG-3′; R: 5′-GGTGGTCCTTCTTGTGCTGC-3′); Twist1 (F: 5′-GGACAAGCTGAGCAAGATTCA-3′; R: 5′-CGGAGAAGGCGTAGCTGAG-3′); GAPDH (housekeeping control; F: 5′-GGAGCGAGATCCCTCCAAAAT-3′; R: 5′-GGCTGTTGTCATACTTCTCATGG-3′). Relative gene expression was calculated via the 2−ΔΔCt method.

## 3 Results and discussion

### 3.1 Identification of T and B cell epitopes in target proteins

**Figure 1:**
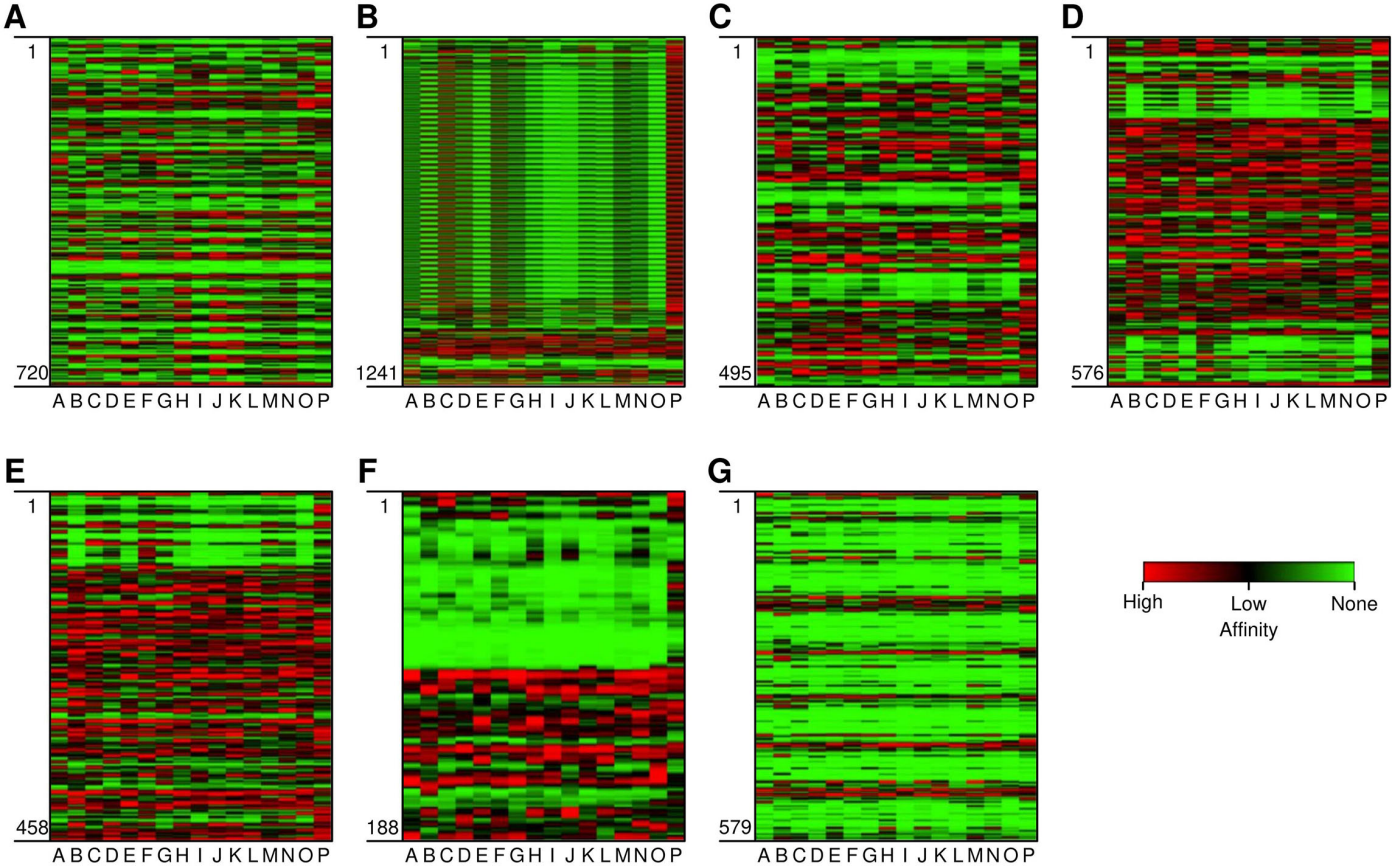
Heat map depicting the affinity of epitopes to different HLA Class II alleles. **(A-G)** are the target proteins: **(A)** Myeloid Zinc Factor, **(B)** Mucin-1, **(C)** Sex determining region Y-box 9, **(D)** Keratin 5, **(E)** Keratin 14, **(F)** Twist related protein-1, and **(G)** Progranulin (GP-88) On Y axis (vertical) are the epitopes from position 1 to last. On the X-axis (horizontal) are the HLA alleles (A-P). The alleles are HLA-DRB1*-01:01, *-02:01, *-03:01, *-04:01, *-07:01, *-08:01, *-09:01, *-10:01*-11:01, *-12:01, *-13:02, *-14:01, *-15:01, DRB3*-02:02, DRB5*-01:01, DPA1*02:02-DPB1*01:01 and HLA-DQA1*-01:02-DQB1*-06:02. Red, black and green region depicts high, low, and non-affinity epitopes to different HLA alleles.

**Figure 2:**
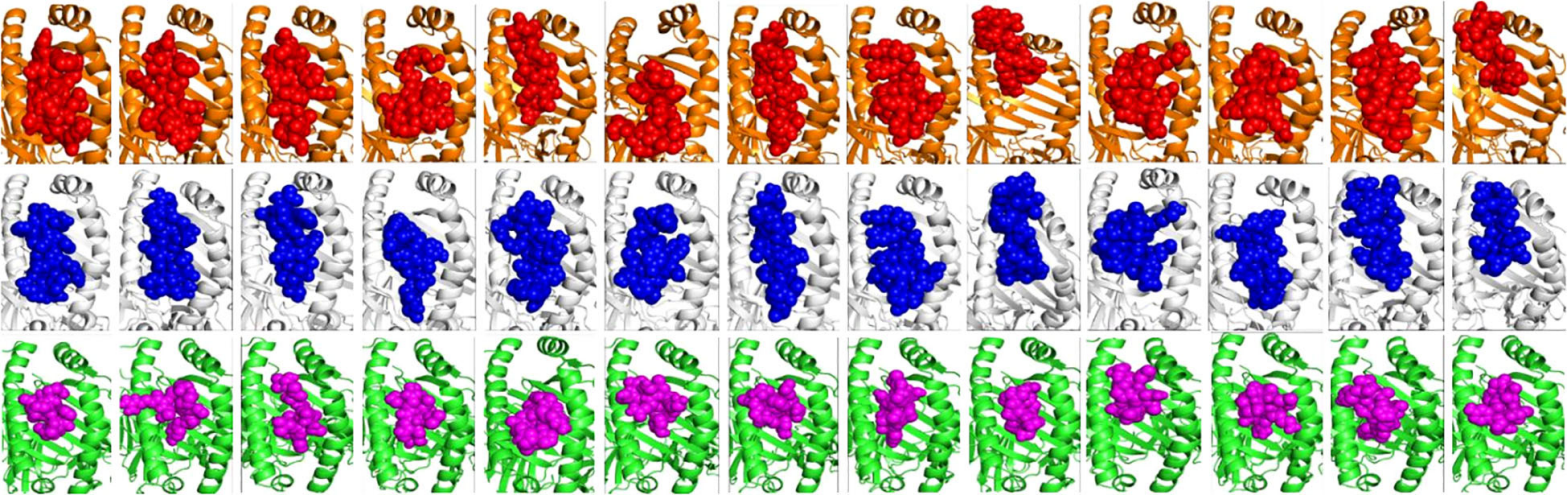
Molecular docking analysis of the finalised HTL and CTL epitopes with HLA Class II and I alleles, respectively. The HLA-DRB1*01:01 & 15:01 alleles are shown in orange and white cartoons and the HTL epitopes in red and blue spheres. The HLA-A*02:01 allele is represented in green coloured cartoon while the Class I epitopes are in magenta colour, respectively. All the epitopes showed high affinity towards HLA alleles with binding in the peptide binding groove (active site) of HLAs (as shown).

**Figure 3:**
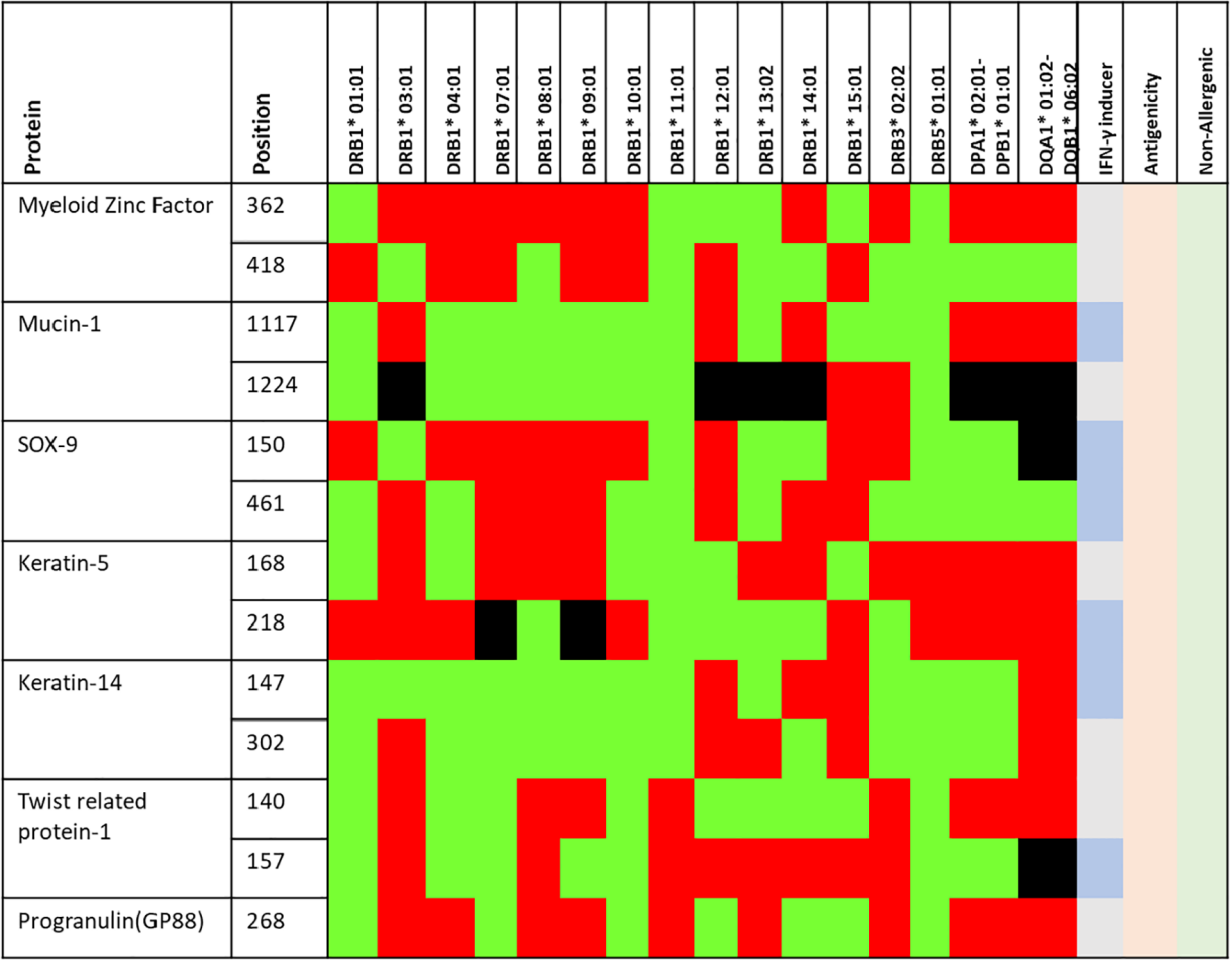
Finally selected epitopes (positions) selected for vaccine construct. The green, red and black region shows high, low and non-binders to different HLA alleles (shown in the table). Further, the epitopes which may induce IFN-γ responses are depicted in slightly darker blue. All the epitopes are antigenic (light orange) and non-allergenic (light green) as predicted by VaxiJen and AllerTop v2.0 servers.

CTL epitopes play a pivotal role in the generation of anti-tumour immunity and in developing therapeutic interventions. CTLs induce programmed cell death (apoptosis) on recognising cancer cells (34). Conversely, HTLs initiate the activation of different immune cells and cytokines through the cascade of reactions. HTLs also contribute to the activation of CTLs. In addition, HTLs also aid in the activation of B cells, which produce antibodies which can neutralise cancer cells or their products (35). Furthermore, HTLs are also indispensable for the generation of memory T cells, thus helping in establishing long-term protective immunity. Therefore, the collaboration between CTLs and HTLs is essential for mounting an effective immune response. Thus, the protein sequences of the designated proteins were subjected to IEDB epitope databases for the identification of CTL and HTL epitopes, targeting a broad spectrum of HLA alleles, widely distributed worldwide. We utilised multiple databases to identify both CTL and HTL epitopes, aiming to improve the accuracy of our prediction method. The epitopes were initially screened based on the affinity to different HLA alleles (**Figure 1**). Subsequently, the selected epitopes underwent a rigorous filtration process through different selection criteria to filter out the best possible epitopes. Initially, the epitopes predicted underwent antigenicity and allergenicity assessment. Then, the extracted antigenic and non-allergenic epitopes were checked for their localisation in the protein. Those present in post-translational modification sites were excluded. Further, the epitopes were checked for promiscuity to different HLA alleles. As promiscuous epitopes have a higher tendency to bind multiple HLA alleles, their inclusion in the vaccine could potentially broaden the population coverage (36). Then the epitopes (HTL & CTL) were modelled and subjected to molecular docking analysis with different HLA alleles in order to find the ones with superior binding affinities (**Figure 2**). The epitopes exhibiting superior binding affinities were selected for subsequent analysis (**Supplementary Table S1**). Previously, various research groups have demonstrated the screening out of the most promising epitopes using a similar strategy (37, 38). IFN-γ has potent antitumour effects, contributing to the production of cytokines. This cytokine may inhibit the growth of cancer cells, induce apoptosis (programmed cell death), and augment the immune response against tumours. In addition, the IFN-γ epitopes stimulate the expression of MHC molecules on the surface of cancer cells. This is essential for the presentation of tumour antigens to T cells, facilitating the recognition and targeting of cancer cells by the immune system (39). Consequently, we also tested the finalised epitopes for their ability to activate IFN-γ cells (**Figure 3**). Upon activation, the B cells undergo differentiation into plasma and memory cells, thus playing a vital role in enduring long‐lasting immunity. Again, we employed multiple servers for the identification of B-cell epitopes. Those predicted by at least two distinct servers and deemed to have antigenic and non-allergenic properties were selected (**Supplementary Table S2**).

### 3.2 Multi-epitope vaccine construction

**Figure 4:**
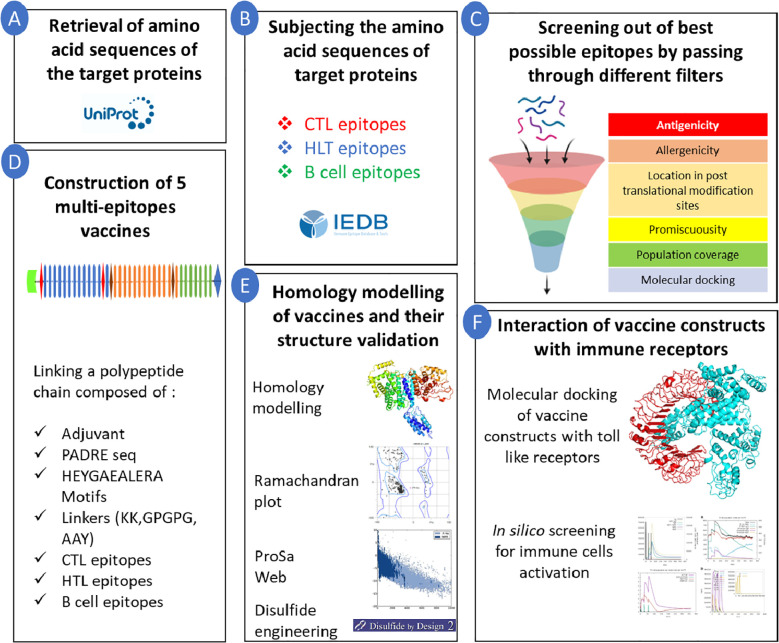
Overall methodology adopted in the present study. Starting from screening of epitopes to finalised vaccine construction. **(A)** Sequence retrieval; **(B)** Sequence subjection; **(C)** Epitope screening; **(D)** 5 multi-epitope vaccine construction; **(E)** Homology modelling; **(F)** Interaction simulation.

The multi-epitope vaccines are capable of inducing the activation of both T and B cells as they are composed of epitopes targeting these cells. In addition, as these vaccines are derived from the antigenic components of single or various proteins, the likelihood of targeting several antigens expressed by cancer cells by such vaccines is feasible. This broadens the immune response, leading to the development of more comprehensive and effective anti-tumour immunity (40). In addition, the chances of immune escape are less in the case of multi-epitope vaccines as they are comprised of several immunogenic epitopes which may trigger complementary immune responses targeting multiple proteins, which may lead to a higher rate of cancer cell recognition, destruction, and tumour regression (41). The vaccines crafted through these innovative strategies hold numerous advantages when compared to classical vaccine design methods. Notably, such vaccines provide extensive coverage across diverse populations and could elicit both cellular and humoral immune responses by incorporating T and B cell epitopes. Additionally, these vaccines are effective even if the target protein is susceptible to mutations as they encompass several epitopes from different proteins. Moreover, such advanced vaccine designs contribute significantly to reducing time and cost compared to traditional methods (40). Hence, the objective of our study was to formulate a vaccine comprising T and B cell epitopes targeting TNBC-associated proteins. The final vaccine sequence was comprised of 13 CD4, 13 CD8, and 7 B‐cell. The chosen epitopes were meticulously selected based on predetermined parameters, including considerations for non-allergenicity, promiscuity, antigenicity, superior affinity towards HLAs (as evidenced by docking scores), and extensive coverage across diverse populations. The overall methodology adopted in the present study from epitope identification to multi-epitope vaccine construction is illustrated in (**Figure 4**).

**Figure 5:**
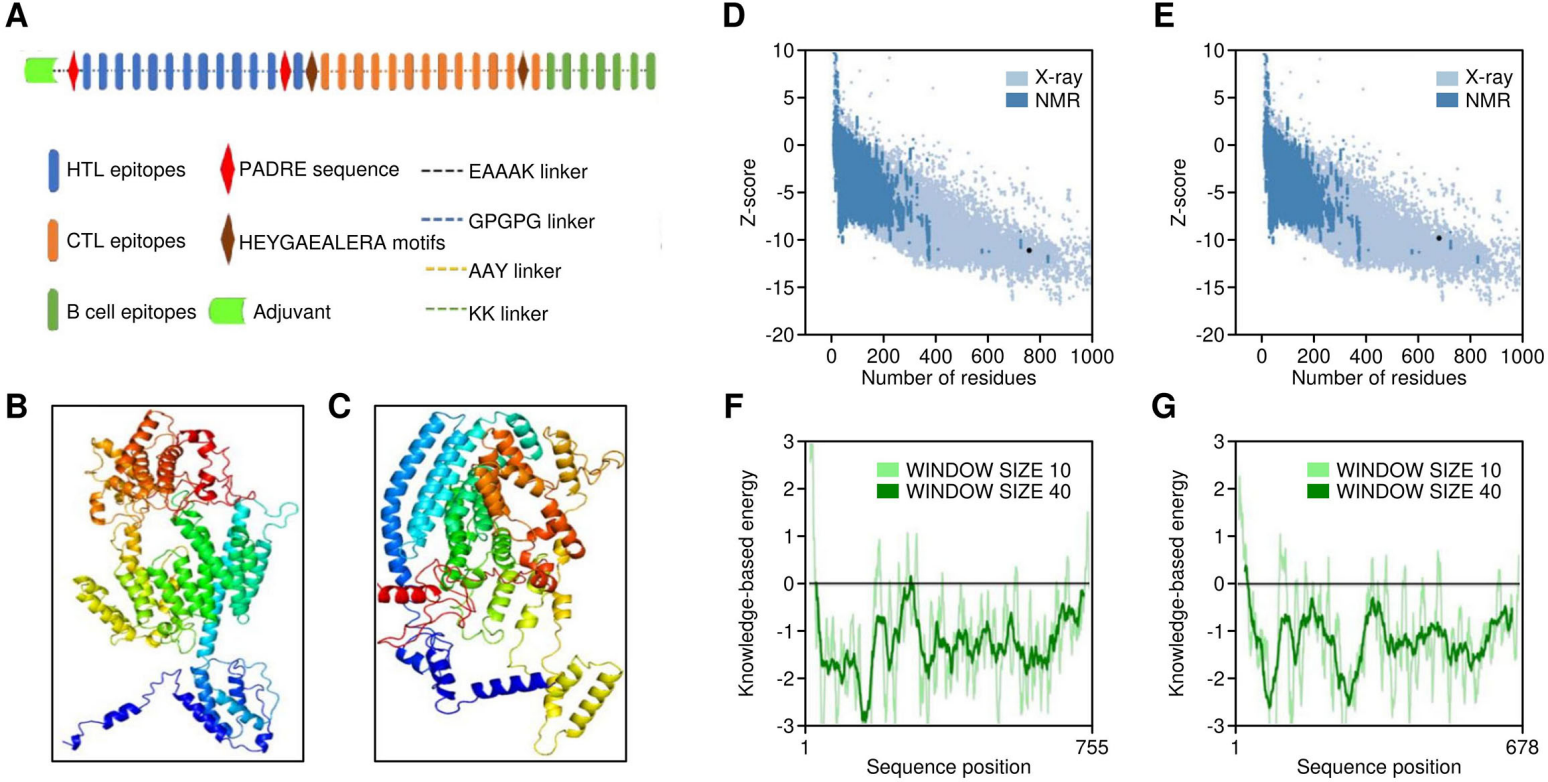
Structural properties of multi-epitope vaccine construct. **(A)** Structural overview of the vaccine featuring different components. **(B, C)** Three-dimensional models of Vaccine 1 and Vaccine 5, depicted in cartoon (rainbow). **(D, E)** Structural quality results for Vaccine 1 3D model by Prosa web, **(F, G)** Structural quality results for Vaccine 5 3D model by Prosa web.

The epitopes corresponding to helper T cells, cytotoxic T cells and B cells were linked using linkers as discussed in the methodology section (**Figure 5**). The GPGPG linkers were successfully introduced as linkers for helper T cell epitopes, restoring their immunogenicity by Livingston et al. (42). These linkers have been widely used to interconnect HTL epitopes within a multi-epitope vaccine and have the ability to induce HLT responses. These linkers also contribute in providing flexibility and to preventing steric hindrance to the epitope chain. The AAY linker is used in interconnecting CTL epitopes, providing structural flexibility for proper folding and facilitating dynamic binding interactions. Moreover, these linkers also function as cleavage sites for the proteasome in mammalian cells, facilitating efficient separation within cells and thereby reducing junctional immunogenicity. Additionally, it has been shown that they can enhance the immunogenicity of the vaccine construct (43). KK linkers are commonly used for interconnecting B-cell epitopes and contribute to immunogenicity as well as spacing and orientation for optimal recognition by B cells and subsequent antibody production (44). A total of 5 vaccine constructs were designed containing different adjuvants: Vaccine 1 (V1) had Granulocyte-macrophage colony-stimulating factor (GM-CSF) as an adjuvant, Vaccine 2 (V2) contained 50s Ribosomal L7/12, Vaccine 3 (V3) contained Interleukin-2 (IL-2), Vaccine 4 (V4) contained Cholera enterotoxin and Vaccine 5 (V5) contained β‐defensin as an adjuvant. All adjuvants were linked to the vaccine constructs via the EAAAK linker GM-CSF functions as a cytokine, promoting the proliferation and differentiation of granulocytes (including neutrophils, eosinophils, and basophils) as well as macrophages. The inclusion of GM-CSF as an adjuvant was aimed at enhancing the overall immune response, promoting the activation and maturation of these immune cells. Including GM-CSF as an adjuvant aimed to boost the overall immune response by fostering the activation and maturation of these immune cells. GM-CSF has also demonstrated the ability to enhance the cytotoxic activity of CTLs with a CD8+ phenotype. This is crucial in the context of TNBC, as an effective CTL response plays a vital role in targeting and eliminating cancer cells (45). Vaccine 2 contained 50s Ribosomal L7/12 as an adjuvant. Ribosomal proteins have immunostimulatory effects by activating innate immune responses. They can engage with pattern recognition receptors (PRRs) on immune cells, resulting in the synthesis of proinflammatory cytokines like TNF-α, IL-1β, and IL-6, and other immune mediators. These also contribute to the activation of T cells (46). Vaccine 3 contained IL-2 as an adjuvant. IL-2 plays a pivotal role as a cytokine in the activation and proliferation of various T cell populations, including cytotoxic T lymphocytes (CTLs). Within the framework of a multi-epitope vaccine, the inclusion of IL-2 could potentially augment the proliferation of antigen-specific T cells, thereby bolstering the immune response targeted against triple-negative breast cancer (TNBC). IL-2 may contribute to enhanced antitumour efficacy by enhancing the cytotoxic potential of T cells. IL-2 has the capability to activate natural killer (NK) cells, aiding in the eradication of cancer cells and potentially complementing the adaptive immune response prompted by a multi-epitope vaccine. IL-2 is involved in modulating the equilibrium between effector T cells and regulatory T cells (Tregs). This balance is critical for achieving an effective and controlled immune response against cancer cells (47, 48). Vaccine 4 contained Cholera enterotoxin as an adjuvant. Cholera enterotoxin has the potential to trigger the activation of antigen-presenting cells (APCs), exemplified by dendritic cells, resulting in improved antigen presentation and subsequent T-cell activation. It has been reported to modulate immune responses, including the promotion of Th2-type responses (49). Vaccine 5 contained β‐defensin as an adjuvant. The adjuvant β‐defensin is widely recognised for its capability to stimulate both innate and adaptive immune responses by interacting with specific immune receptors, including Toll-like receptors (TLRs) and CCR6. This process includes the attraction of T cells and nascent dendritic cells to the infection site (50), indicating its potential as a promising adjuvant.

Additionally, a PADRE sequence was also implemented at the N terminus. It is known to induce HTL responses and have an affinity towards numerous HLA Class II alleles (51). Furthermore, linkers consisting of HEYGAEALERA motifs were utilised to connect cytotoxic T‐cell epitopes. These linkers play a crucial role in improving the presentation of epitopes by providing specific sites for both lysosomal and proteasomal-mediated cleavages, specifically at positions A5‐E6, Y3‐G4, A7‐L8, L8‐E9, and R10‐A11 (52). The vaccine sequence underwent random reshuffling of epitopes, resulting in five different sequences. These sequences were then subjected to homology modelling to attain the optimal configuration (**Figures 5B**). The models were subjected to Ramachandran plot analysis in order to identify the model with the best structural configuration (**Supplementary Figure S1**). Moreover, the completed model underwent refinement using the GalaxyRefine server, a well-regarded tool for enhancing the overall structure of 3D models (53), utilising CASP10-associated refinement techniques. It is recognised as one of the premier servers for enhancing the protein’s global and local structural configuration. The quality of the finally generated models was checked by ProSA web (**Figures 5D**) and Ramachandran Plots. The evaluation of population coverage in terms of HLA Class I and Class II alleles targeted by the epitopes finally selected revealed 80.71% and 93.36%, respectively. Moreover, the global population reached 98.72% on the inclusion of CTL and HTL epitopes in the vaccine construct (**Supplementary Figure S2**). The amino acid sequences of all five finalised vaccine constructs were observed to be immunogenic (as revealed by VaxiJen server) and nonallergenic (as revealed by AlgPred and AllerTOP v2.0 servers). Further, the modelled vaccine constructs underwent disulfide engineering to enhance thermos-stability, employing the Design 2 server. By this process, the entropy associated with the conformational changes of the unfolded state of the protein is reduced.

### 3.3 Interaction of vaccine constructs with toll-like receptors

**Figure 6:**
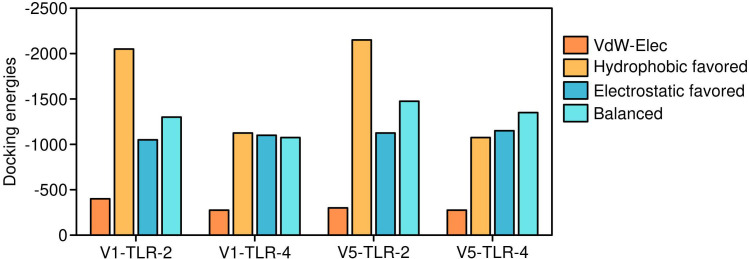
Docking energies of Vaccine 5 and Vaccine 2 constructs. The detailed interaction energies of Vaccine 5 and Vaccine 4 with toll-like receptors.

**Figure 7:**
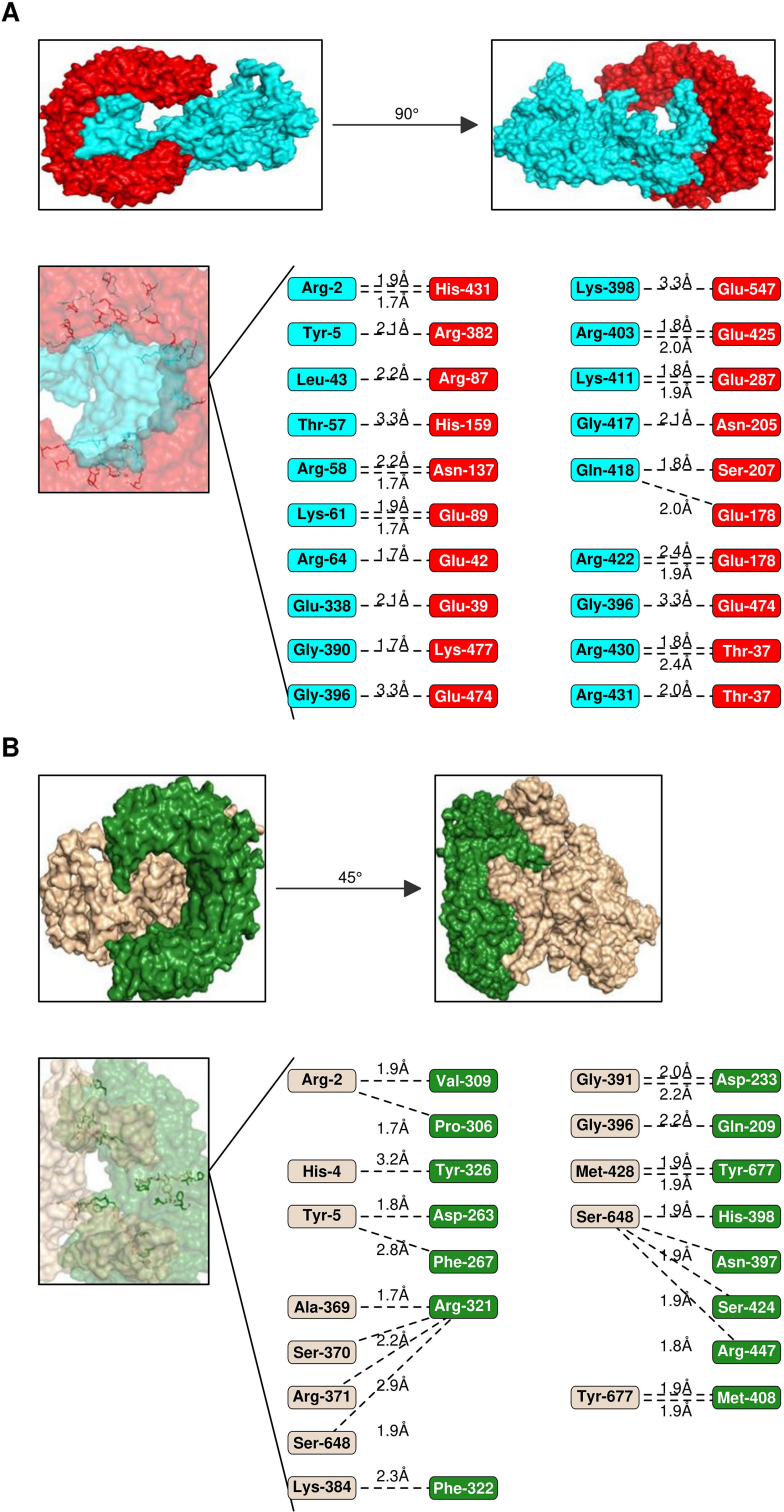
Interaction analysis of Vaccines 5 & 2 with TLRs. **(A)** Interaction pattern of Vaccine 5 (Cartoon- cyan) with TLR-2 (cartoon, red). The amino acid residues engaged in the interactions are represented below along with H-bond lengths. **(B)** Interaction pattern of Vaccine 1 (Cartoon- wheat colour) with TLR-2 (cartoon, forest green).

The efficacy of a vaccine or drug depends on its stable and robust interaction with its receptor. Thus, all five vaccine constructs were docked with TLR-2 & 4. The V5 vaccine construct showed the highest affinity with toll-like receptors, followed by V1, V2, V4 and V3 constructs (**Figures 6**, **7**). Earlier, several research groups have adopted a similar kind of strategy for investigating the interactions between multi-epitope vaccines and immune receptors (54).

### 3.4 Immune simulation

**Figure 8:**
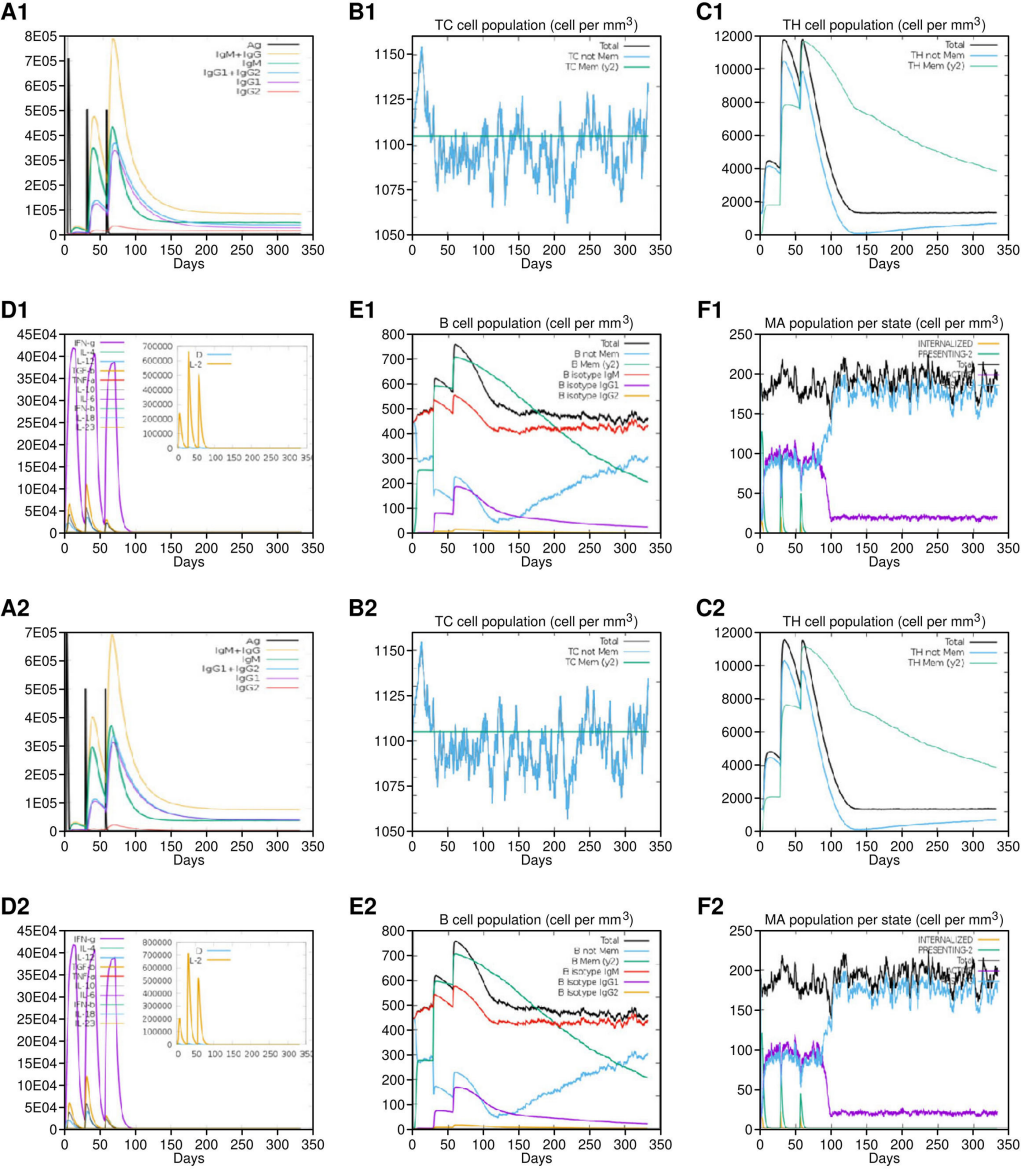
Immune simulation results of Vaccine 1 **(A1-F1)** and Vaccine 5 **(A2-F2)**. Both vaccines showed almost similar immune activation. **(A1/A2)** Production of vaccine-specific antibodies upon administration. **(B1/B2)** Cytotoxic T cell proliferation in response to vaccine. **(C1/C2)** Helper T cells proliferation in response to vaccine. **(D1/D2)** Spike in interleukins in response to vaccine. **(E1/E2)** B cells response. **(F1/F2)** Macrophages activation.

**Figure 9:**
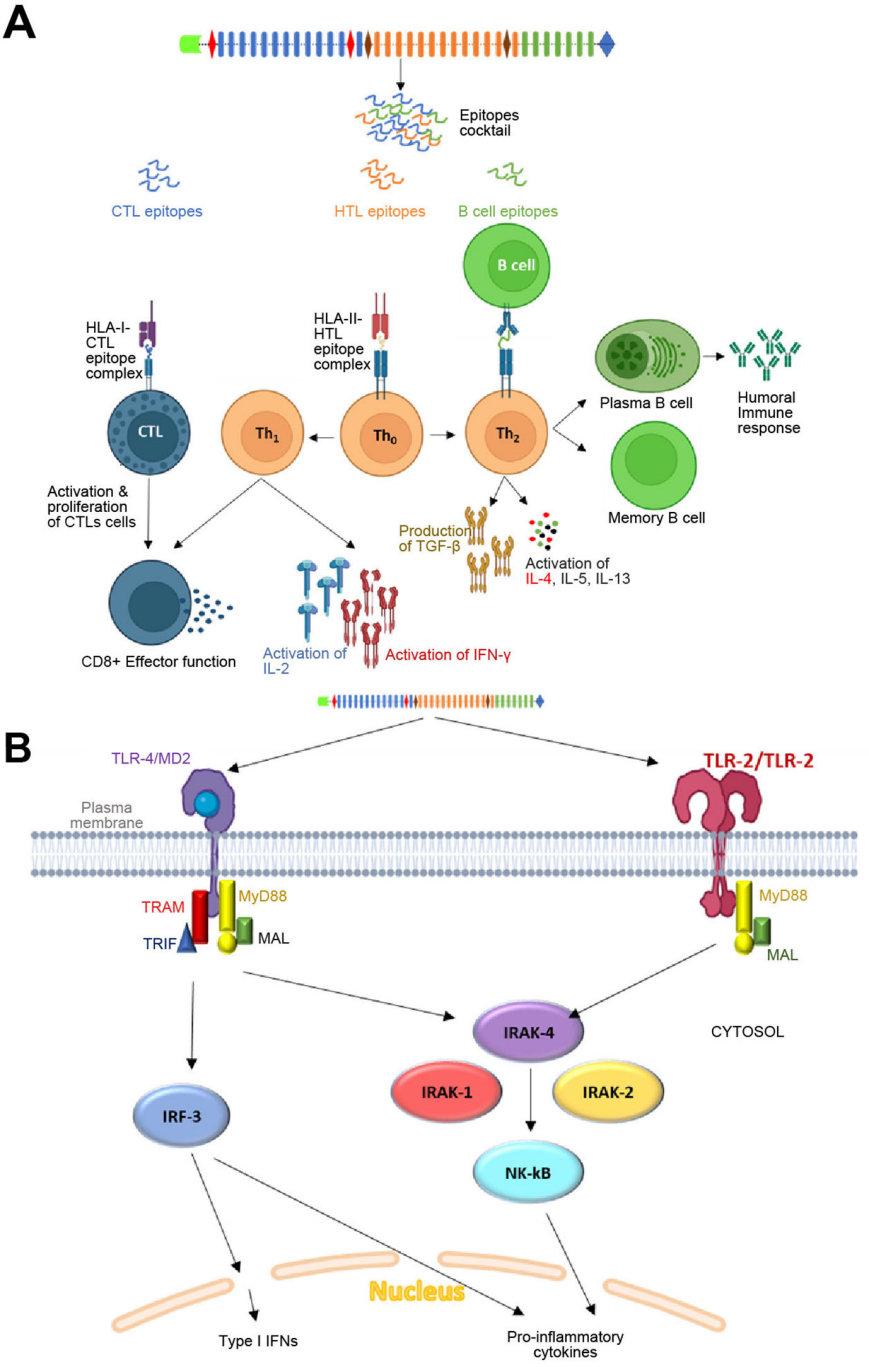
Mechanism of action of the proposed vaccine in host: **(A)** Adaptive immune response by the vaccine construct. The CTL, HTL and B cell epitopes linked by different linkers will be processed in Antigen-presenting cells (APCs). The CTL epitopes will be presented by HLA-Class I alleles to cytotoxic T cells, which may further lead to activation of the effector function of Tc cells. Similarly, HTL epitopes will be presented by HLA-Class II alleles to Th_o_ cells, which will subsequently undergo differentiation into Th_1_ and Th_2_ cells. Th_1_ will initiate the activation of IL-2 and IFN-γ cells through the cascade of reactions. The Th_2_ cells will lead to activation and proliferation of TGF-β, IL-4, IL-5 and IL-13. The Th_2_ cell-mediated cytokines may further lead to the activation and differentiation of plasma B cells and memory B cells for antibodies generation. The vaccine-specific antibodies generated may further the target proteins through two mechanisms: one involving complement-dependent cytotoxicity (CDC) and the other antibody-dependent cell-mediated cytotoxicity (ADCC). **(B)** Innate immune response by the vaccine construct in host: TLR-2 homodimer (employing 2 adapters as depicted in the figure) and TLR-4 (employing 4 adapters as depicted in the figure) expressed on the surface of antigen-presenting cells (APCs) will initiate a cascade of immune reactions, leading to the activation of Type-1 interferons and proinflammatory cytokines.

The amino acid sequences of vaccine constructs V1 and V5 were further subjected to *in silico* immune simulations to investigate their capabilities to generate an effective immune response. The parameters for carrying out the simulation included 1000 steps, three injections administered at 1-, 84-, and 168-time lapses, which corresponded to 1, 28, and 56 days, respectively. Other parameters like simulation volume (10) and random seed were not altered and ran at default. Both V1 and V5 vaccines showed almost similar kind of immune responses as revealed by *in-silico* immune simulation findings. There was an initial increase in immunoglobulin M (IgM) levels, followed by heightened levels of IgG and its subclasses, accompanied by a reduction in antigen concentrations. The vaccine exhibited its ability to induce robust and effective immunity, as evidenced by elevated levels of T cells, macrophages, IFN‐γ, interleukin‐2, as well as activated and memory B cells. The capacity to produce robust and efficient immunity by the vaccine was suggested by increased levels of T & B cells, macrophages, IFN‐γ and IL-2 (**Figure 8**). In addition, we also attempted to explain an overall mechanism of the proposed vaccine construct in host, how it may modulate both innate and adaptive immune response in the host (**Figure 9**).

### 3.5 qPCR confirms target gene downregulation

The *in vitro* qPCR data substantiate the reliability of our computational pipeline, demonstrating that the selected vaccine epitopes can effectively modulate the expression of critical transcription factors implicated in TNBC progression. Specifically, exposure of MDA-MB-231 cells to the vaccine epitopes resulted in significant downregulation of MZF-1 (2.1-fold, *p* = 0.008), SOX-9 (1.8-fold, *p* = 0.012), and Twist1 (2.4-fold, *p* = 0.003), consistent with our *in-silico* predictions (**Table 1**). These transcription factors are well-established regulators of epithelial-mesenchymal transition, proliferation, and metastasis in triple-negative breast cancer (55). The observed transcriptional suppression indicates a potential mechanism by which the designed epitopes may interfere with oncogenic signalling cascades. While our computational models predicted strong binding affinities to HLA molecules and interaction with innate immune receptors such as TLRs, the current *in vitro* findings provide the first layer of biological validation. Future studies will aim to build on these results by incorporating functional immune assays, such as ELISpot for IFN-γ secretion, as well as *in vivo* tumour models, to evaluate the full immunotherapeutic potential of the epitope-based vaccine.

**Table 1:** Transcriptional response of MDA-MB-231 cells to vaccine epitope exposure as determined by qPCR.

Gene	Relative Expression (Fold Change)	p-value	Regulation Direction	Notes
MZF-1	0.48 (↓ 2.1-fold)	0.008	Downregulated	Significant
SOX-9	0.56 (↓ 1.8-fold)	0.012	Downregulated	Significant
Twist1	0.42 (↓ 2.4-fold)	0.003	Downregulated	Highly significant

## 4 Conclusion

The present study offers a comprehensive approach to the identification of immunodominant epitopes and the further development of a multiepitope vaccine against TNBC. The key proteins, as discussed above in the introduction section, were selected strategically, aiming to target multiple aspects of tumour growth, progression and metastasis. The rigorous immune-informatics pipeline design led to the successful identification and screening of the potential epitopes capable of activating CTL, HTL and B cell responses. The criteria for evaluating the epitopes ensured the selection of the most promising epitopes that may be considered for inclusion in multi-epitope vaccines. The molecular docking analysis revealed notable affinity between the toll-like receptors and the vaccine model, suggesting potential immunogenic interactions. Furthermore, the robust and sustained immune responses by the vaccine constructs with elevated levels of different immune cells were supported by *in silico* immune simulation findings. The present study presents a multi-epitope peptide vaccine-based approach that targets TNBC-specific oncogenic proteins. Unlike the checkpoint inhibitors, which are known to act broadly, the presently proposed vaccine is based on eliciting a T cell-based immune response against tumour-associated antigens. Further, the *in-vitro* validation showed signification downregulation of MZF-1, SOX-9, Twist1 proteins, indicating the upper hand by direct functional impact, which current immunotherapies do not measure. Furthermore, the efficacy of immune-based therapies is also hindered by tumour microenvironment in TNBC, which is rich in immunosuppressive cells. However, the presented multi-epitope vaccine enhances antitumour immunity, potentially modifying the TME by targeting important oncogenic drivers involved in EMT. Therefore, the proposed vaccine presents targeted immunotherapy, broader applicability regardless of BRCA/PD-L1 status, lower toxicity, and a specific mechanism-based intervention.

## Data Availability

The datasets presented in this study can be found in online repositories. The names of the repository/repositories and accession number(s) can be found in the article/**Supplementary Material**.

## Supplementary material

Supplementary Table 1 Docking energies of HTL epitopes from different proteins with HLA class II alleles (DRB1*01:01//DRB1*15:01).

Supplementary Table 2 Predicted B cell epitopes in the target proteins.

Supplementary Figure 1 Ramachandran plot of Vaccine 1 and Vaccine 5.

Supplementary Figure 2 Worldwide population coverage by the epitopes included in the vaccine construct.

Supplementary Table 3 qPCR Analysis of Gene Expression in MDA-MB-231 Cells Following Epitope Exposure.
