# Supplementary material for: In silico design and in vitro validation of a multi-epitope peptide vaccine targeting triple-negative breast cancer
Source: Front Oncol. 2025 Jun 23;15:1611991. doi: 10.3389/fonc.2025.1611991 (PMC12229876; doi:10.3389/fonc.2025.1611991)
Supplement: Supplementary Table 1 — Docking energies of HTL epitopes from different proteins with HLA class II alleles (DRB1*01:01//DRB1*15:01). [file DataSheet1.docx]

**Supplementary Files**

**Table S 1:** Docking energies of HTL epitopes from different proteins with HLA class II alleles (DRB1*01:01 // DRB1*15:01).

| **Protein** | | | | |
| --- | --- | --- | --- | --- |
| **Myeloid Zinc Factor** | | | | |
| **Position** | **B** | **E** | **H** | **V** |
| 47 | -753.5 //-724.8 | -754.3 // -754.9 | -1061.6 // -117.7 | -139.4 // -150.6 |
| 361 | -823.3 //-824.7 | -889.1 // -884.1 | -1121.2 // -1263.6 | -169.2 // -163.7 |
| 362 | -725.7 //-762.4 | -834.8 // -795.7 | -867.5 // -956.6 | -188.6 // -187.5 |
| 418 | -842.5 //-866.3 | -889.1 // -895.3 | -1073.5 // -1162.9 | -162.9 // -151.8 |
| **Mucin-1** | | | | |
| 968 | -612.3 //-654.7 | -672.3 // -674.7 | -837.3 // -819.7 | -136.6 // -129.6 |
| 970 | -880 // -764.6 | -950.9 // -790.9 | -1078 // -949.3 | -132.5 // -119.4 |
| 1117 | -686.7 //-722.3 | -712.9 // -734.5 | -848.4 // -785.4 | -135.6 // -149.5 |
| 1224 | -611 // -586.1 | -631.4 // -582 | -776.6 // -674.8 | -140.7 // -144.1 |
| 1225 | -600 // -590.8 | -614.5 // -669.3 | -659 // -696.8 | -124.7 // -151.4 |
| 1226 | -669.8 // -599.5 | -712.9 // -625.9 | -809.4 // -725.4 | -145.6 // -140.9 |
| 1227 | -633.6 // -608.0 | -570.3 // -658.5 | -761.9 // -795.4 | -129.4 // -148.9 |
| **SOX-9** | | | | |
| 150 | -663.6 // -645.1 | -657.8 // -700.8 | -869.8 // -864.2 | -143.7 // -153.4 |
| 201 | -724.1 // -643.1 | -698 // -665.8 | -1014.3 // -884.5 | -126.5 // -142.7 |
| 202 | -619.7 // -614.3 | -642 // -632.9 | -841.3 // -796 | -134.6 // -139.3 |
| 461 | -959.8 // -1028.8 | -981.4 // -1053.8 | -1257.8 // -1455.6 | -144.1 // -148.6 |
| **Keratin-5** | | | | |
| 167 | -589.2 // -575.6 | -662.3 // -545.0 | -722.3 // -669.8 | -183.6 // -164.1 |
| 168 | -750 // -769.4 | -789.1 // -789.6 | -1097.1 // -913.4 | -157.5 // -160.3 |
| 183 | -578.1 // -615.4 | -585.1 // -642.1 | -811 // -782.5 | -145 // -143.1 |
| 184 | -658.7 // -649.4 | -634 // -672.7 | -839.4 // -752.2 | -155.9 // -156.5 |
| 185 | -586.7 // -581.8 | -684.7 // -599.8 | -715.2 // -668.2 | -157.4 // -159.1 |
| 218 | -840.2 // -806.2 | -864.7 // -802.9 | -1108.7 // -1098.2 | -124 // -141.8 |
| 220 | -782.7 // -797.4 | -823.7 // -801.6 | -1138.2 // -1150.9 | -130.4 // -140.7 |
| 360 | -715 // -777.2 | -792.3 // -713.2 | -1097 // -1076.1 | -156.7 // -141.8 |
| 361 | -678.2 // -643.8 | -693.3 // -700.3 | -854.3 // -845.7 | -120.1 // -123.8 |
| 464 | -606.6 // -618.8 | -561.9 // -577.1 | -852.7 // -866.6 | -121.8 // -121.4 |
| **Keratin-14** | | | | |
| 146 | -742.8 // -797.9 | -803.8 // -871.3 | -1054.3 // -1174.5 | -184.5 // -183.9 |
| 147 | -820.1 // -803.8 | -857.2 // -874.9 | -1200.8 // -1125.8 | -130.2 // -181.3 |
| 149 | -785.1 // -683.1 | -798.4 // -686.1 | -903.6 // -874.9 | -158.9 // -130.1 |
| 183 | -686.6 // -652.5 | -723.9 // -665.6 | -850.6 // -859.1 | -116.9 // -123.8 |
| 184 | -660.9 // -615.6 | -659.9 // -613.9 | -929.4 // -839.3 | -101.6 // -100.4 |
| 185 | -635.0 // -625.6 | -630.9 // -614.7 | -950 // -900.3 | -110.1 // -118.6 |
| 301 | -782.7 // -871.4 | -760.3 // -834.0 | -1166.5 // -1303.1 | -121.1 // -122.7 |
| 302 | -883.6 // -893.4 | -843.2 // -851.3 | -1254.5 // -1297.2 | -176.2 // -146.7 |
| 303 | -827.3 // -730.1 | -755.2 // -678.9 | -1028.4 // -1121.4 | -112.5 // -124.2 |
| 396 | -811.7 // -742.3 | -808.7 // -798.3 | -1081.7 // -1027.4 | -131.2 // -148.1 |
| 409 | -633.4 // -659.8 | -615.4 // -613.4 | -867.3 // -811 | -128.5 // -123.4 |
| **Twist related protein-1** | | | | |
| 97 | -644.1 // -647.7 | -666.8 // -659.7 | -792.2 // -857.1 | -124.5 // -118.0 |
| 139 | -485.8 // -472.5 | -528.0 // -565.8 | -592.3 // -571 | -154.1 // -152.4 |
| 140 | -558.4 // -560.2 | -670.6 // -599.7 | -738 // -764.3 | -195.1 // -195.9 |
| 157 | -641.6 // -636.7 | -596.4 // -583.1 | -920.2 // -879.2 | -120.1 // -128 |
| **Progranulin (GP88)** | | | | |
| 267 | -636.0 // -631.5 | -631.9 // -628.5 | -827.3 // -913.6 | -103.9 // -110.3 |
| 268 | -678.4 // -637.4 | -707.0 // -627.8 | -996.3 // -930.1 | -115.7 // -183.0 |

**Table S 2:** Predicted B cell epitopes in the target proteins.

| **Myeloid Zinc Factor** | |
| --- | --- |
| 12 | APPEDEGPVMVKLEDS |
| 344 | GRPSTGGGVVRGGRCD |
| 350 | GGVVRGGRCDVCGKVF |
| **Mucin-1** | |
| 1139 | VSVSDVPFPFSAQSGA |
| 1225 | DRSPYEKVSAGNGGSS |
| **Sox-9** | |
| 70 | PVCIREAVSQVLKGYD |
| **Keratin-5** | |
| 298 | DEINFMKMFFDAELSQ |
| 88 | GAGAGGGYGFGGGAGS |
| 473 | LLEGEECRLSGEGVGP |
| 235 | GERGRLDSELRNMQDL |
| **Keratin-14** | |
| 157 | AEIKDYSPYFKTIEDL |
| 326 | SGKSEISELRRTMQNL |
| 284 | LNEMRDQYEKMAEKNR |
| 21 | GGGIGGGSSRISSVLA |
| 63 | CGLGGGYGGGFSSSSS |
| 57 | FSSGGACGLGGGYGGG |
| 173 | RNKILTATVDNANVLL |
| **Twist related protein-1** | |
| 57 | GGGVGGGDEPGSPAQG |
| 79 | AGCGGGGGAGGGGGSS |
| **Progranulin** | |
| 16 | AGTRCPDGQFCPVACC |
| 70 | GHSCIFTVSGTSSCCP |
| 443 | GCDQHTSCPVGQTCCP |


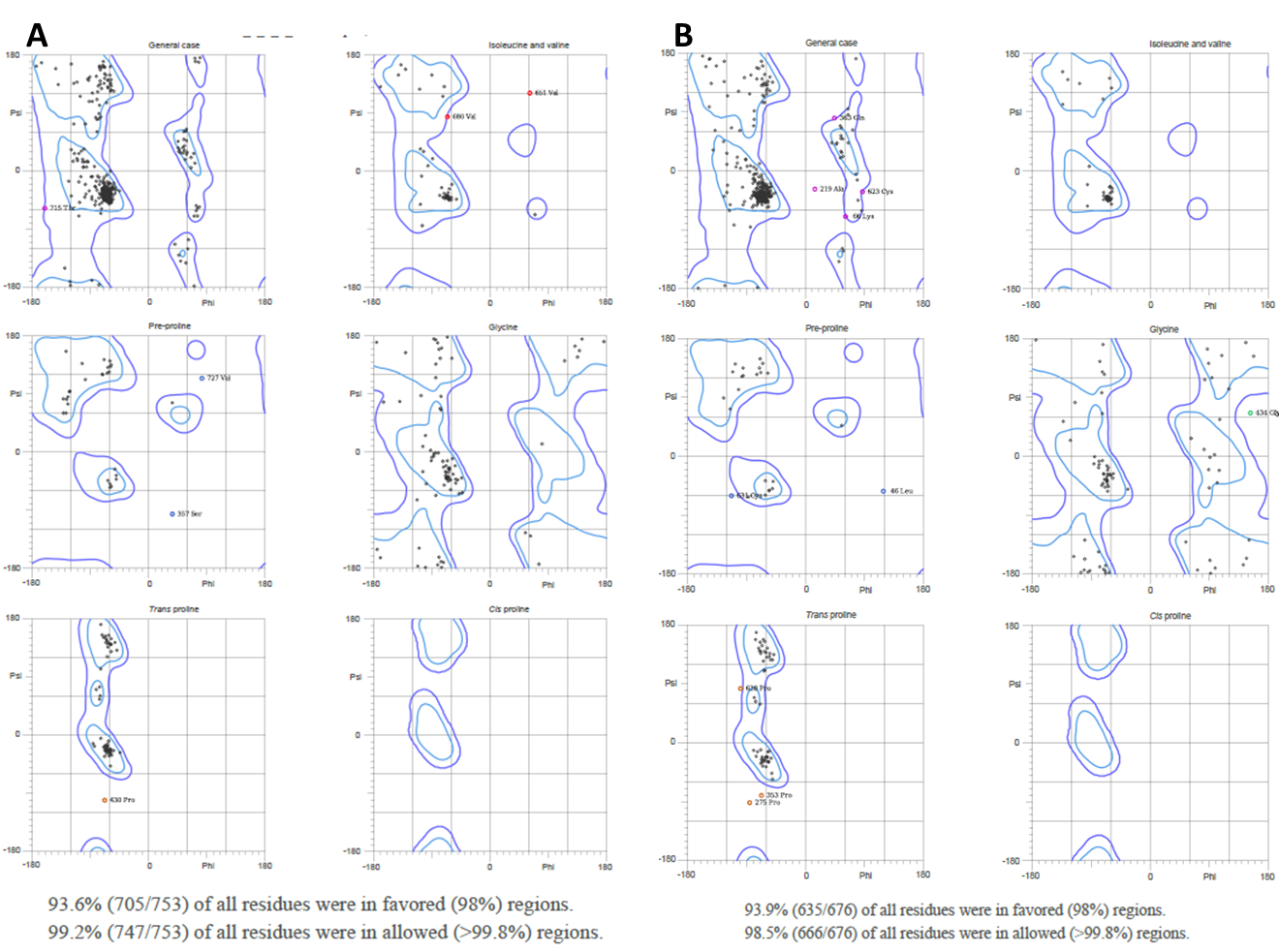


**Fig. S 1:** Ramachandran plot of Vaccine 1 and Vaccine 5.


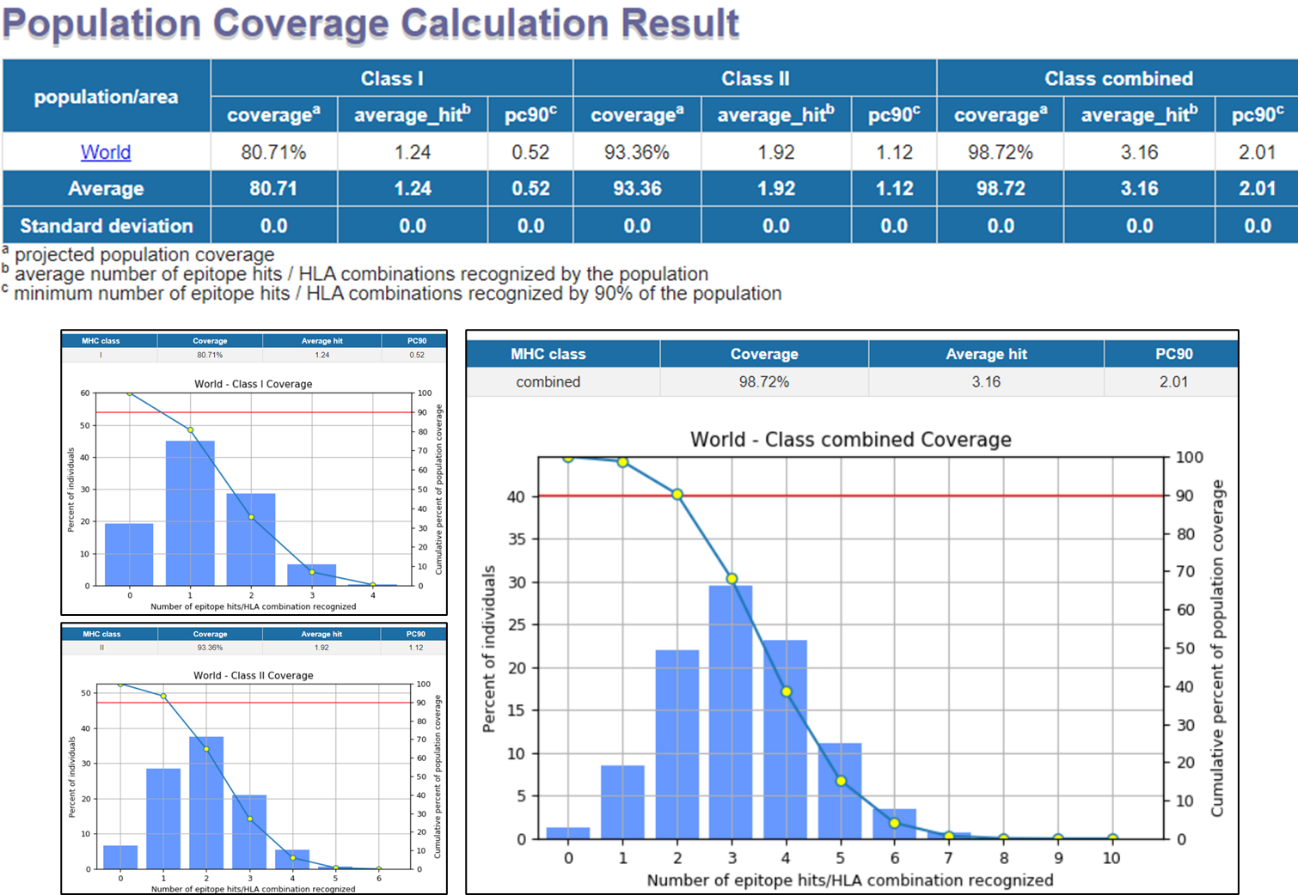


**Fig. S 2:** Worldwide population coverage by the epitopes included in the vaccine construct.

| **S Table 3:** **qPCR Analysis of Gene Expression in MDA-MB-231 Cells Following Epitope Exposure** | | | | | | |
| --- | --- | --- | --- | --- | --- | --- |
| **Gene** | **Mean ΔΔCt** | **Fold Change (2^-ΔΔCt)** | **Log2 Fold Change** | **p-value** | **Regulation** | **Notes** |
| MZF-1 | 1.07 | 0.48 | -1.06 | 0.008 | Down | ~2.1-fold downregulation, significant |
| SOX-9 | 0.83 | 0.56 | -0.83 | 0.012 | Down | ~1.8-fold downregulation, significant |
| Twist1 | 1.27 | 0.42 | -1.24 | 0.003 | Down | ~2.4-fold downregulation, highly significant |

Note; Fold change values (2^-ΔΔCt) were calculated assuming ΔΔCt = log2(1/Fold Change). Log2 fold change is the log base 2 of Fold Change.
